# Murine hindlimb ischemia models: a narrative review

**DOI:** 10.3389/fcvm.2025.1700963

**Published:** 2026-01-09

**Authors:** Yongkang Zhang, Hongcheng Du, Qingzhi Ran, Jiaruo Xu, Xinyi Tang, Yuzhen Wang, Junlin Deng, Yemin Cao

**Affiliations:** 1 Innovation Research Institute of Traditional Chinese Medicine, Shanghai University of Traditional Chinese Medicine, Shanghai, China; 2 Diagnosis & Treatment Center of Vascular Diseases, Shanghai TCM-Integrated Hospital, Shanghai University of Traditional Chinese Medicine, Shanghai, China; 3 Department of Traditional Surgery, Shuguang Hospital Affiliated to Shanghai University of Traditional Chinese Medicine, Shanghai, China; 4 Guang’anmen Hospital, China Academy of Chinese Medical Sciences, Beijing, China

**Keywords:** animal disease models, hindlimb ischemia, ischemia-reperfusion, murine models, peripheral arterial disease

## Abstract

The murine hindlimb ischemia model is a classical experimental platform for studying peripheral arterial disease (PAD). It primarily includes three categories: acute ischemia models, subacute-to-chronic ischemia models, and ischemia-reperfusion models. Acute ischemia models are the most widely applied, with relatively well-established methodologies. They are typically induced by ligation or electrocauterization, but other approaches such as interventional embolization, photochemical thrombosis, physical injury, chemical injury, and embolization with traditional Chinese medicine-derived microparticles have also been employed. Subacute-to-chronic ischemia models, including the Ameroid constrictor, intravascular suture, anticoagulant silicone tube, and loop techniques, better reflect the pathophysiology of chronic PAD but have not yet been standardized for broad use. Ischemia-reperfusion models, as specialized interventional variants, are increasingly studied in the context of revascularization-related injuries. This review summarizes the methodologies, advantages, and limitations of the above models, and highlights factors influencing model establishment and experimental outcomes, aiming to provide a comprehensive reference for future research using murine hindlimb ischemia models.

## 1 Introduction

Peripheral arterial disease (PAD) is an ischemic disorder characterized by arterial stenosis or occlusion secondary to atherosclerosis, involving extracranial carotid, upper limb, renal, mesenteric, and lower limb arteries (1). In most of the literature, PAD refers specifically to lower limb arterial disease, which is also the focus of this review. Globally, over 200 million individuals are affected by PAD, presenting clinically with limb pain, pallor, coldness, numbness, intermittent claudication, rest pain, or even gangrene. These symptoms severely impair quality of life and threaten survival. Current therapeutic strategies mainly include pharmacologic interventions and revascularization procedures (2).

Preclinical validation in animal models is essential prior to clinical application of new therapeutic approaches. Establishing a reliable and appropriate PAD model provides the foundation for translational studies. While rabbits, pigs, and sheep have all been employed in hindlimb ischemia research (3), murine models are most widely used (4). These models can be categorized into acute ischemia, subacute-to-chronic ischemia, and ischemia-reperfusion models. Acute models are usually induced by ligation or electrocauterization, interventional or photochemical embolization, physical or chemical injury, or embolization with traditional Chinese medicine (TCM)-derived microparticles. They are technically mature, rapidly induce ischemia, and are associated with relatively low operative risk (5). However, the ischemic period is often short, differing substantially from the progressive nature of chronic occlusive PAD in humans. Subacute-to-chronic ischemia models, such as the Ameroid constrictor, intravascular suture, anticoagulant silicone tube, and loop methods, provide a more accurate simulation of chronic ischemia, though none has yet become widely adopted. With the advancement of interventional therapies—including balloon angioplasty, drug-coated balloons, stent implantation, and debulking techniques—the need to study ischemia-reperfusion (I/R) injury has also emerged. In these models, careful attention must be paid to the duration of ischemia and the associated reperfusion injury.

A comprehensive literature search was conducted using the electronic databases PubMed, Web of Science, and Google Scholar for relevant articles published up to March 2025. The search terms included: (“murine” OR “mouse” OR “rat”) AND (“hindlimb ischemia” OR “hind limb ischemia”) AND (“model” OR “modeling”) AND (“peripheral arterial disease”). The reference lists of retrieved articles were also manually screened to identify additional relevant studies. Inclusion criteria focused on original research articles and reviews that detailed the establishment, application, or evaluation of murine hindlimb ischemia models. Studies involving other animal species or not primarily focused on modeling methodology were excluded. The objective of this review is to summarize current methodologies for establishing murine hindlimb ischemia models, compare their advantages and disadvantages, and analyze critical factors that may affect model validity and experimental outcomes. These insights may help guide researchers in selecting or optimizing models for future studies.

## 2 Anatomy of the murine hindlimb arteries

**Figure 1:**
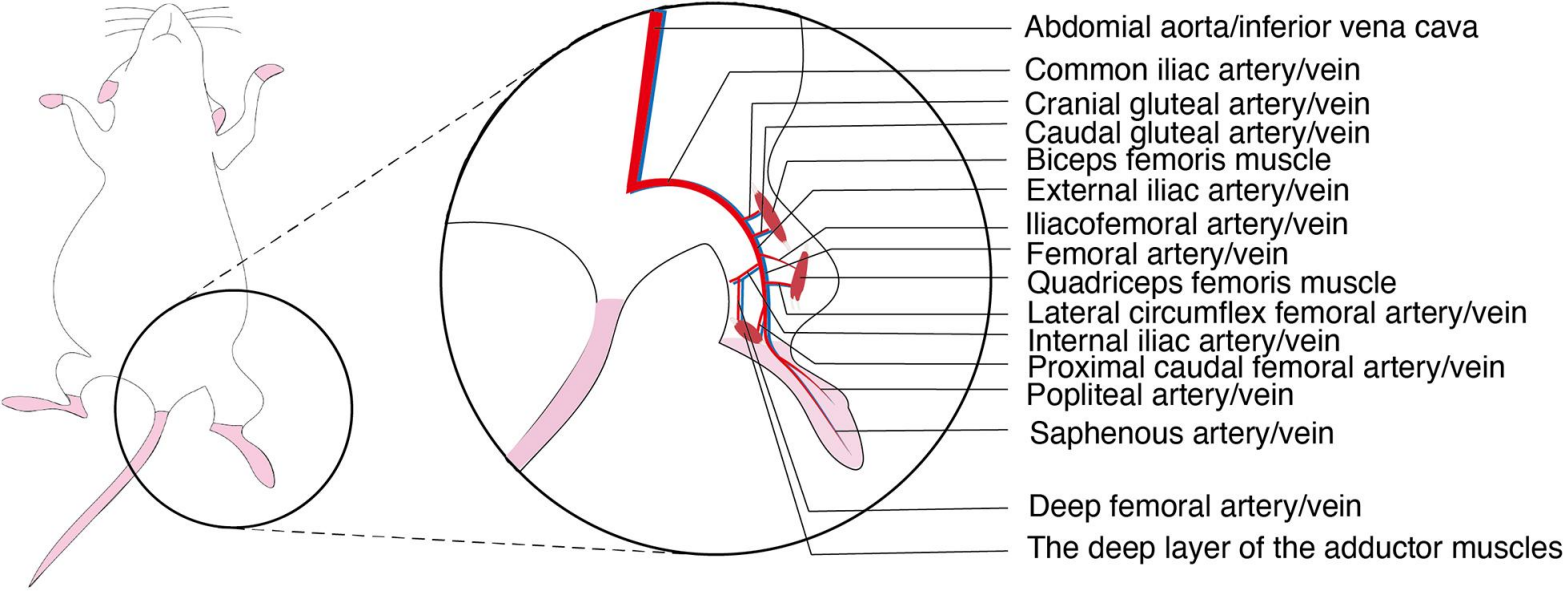
Anatomical diagram of the arteries and veins of the hindlimbs of murine.

The abdominal aorta in mice gives rise to the left and right common iliac arteries as well as the median sacral artery. Distally, the common iliac artery branches sequentially into the cranial gluteal artery, caudal gluteal artery, iliacofemoral artery, external iliac artery and internal iliac artery. The iliacofemoral artery originates from the distal segment of the common iliac artery and lies dorsal to the internal iliac artery. The external iliac artery serves as the major arterial supply to the hindlimb; after passing beneath the inguinal ligament, it continues as the femoral artery. Distally, the femoral artery gives rise to the lateral circumflex femoral artery (anterior femoral artery) and the proximal caudal femoral artery. At the proximal level of the knee joint and distal to the superficial epigastric artery, the femoral artery bifurcates into the popliteal artery and the saphenous artery. The popliteal artery traverses transversely through the popliteal fossa between the quadriceps femoris and the adductor muscles. Near its origin, the popliteal artery gives off the proximal medial genicular artery, which supplies the distal quadriceps femoris. The cranial and caudal gluteal arteries supply the biceps femoris muscle, while the iliacofemoral artery, lateral circumflex femoral artery, and proximal medial genicular artery supply the quadriceps femoris muscle. The deep femoral artery, proximal caudal femoral artery, and small branches from the saphenous and popliteal arteries supply the deep layer of the adductor muscles. The saphenous artery and the distal branches of the popliteal artery repeatedly branch to provide arterial supply to the foot (Figure 1) (6).

## 3 Acute ischemia model

The acute hindlimb ischemia model is primarily established by rapidly interrupting arterial blood flow in the hindlimb through approaches such as ligation, electrocoagulation, interventional embolization, photochemical thrombosis, physical injury, chemical injury, or embolization with TCM microparticles (Table 1). Considerable variability often arises from the use of different modeling techniques, as well as from variations within the same technique. Therefore, it is essential to clearly define the specific procedures for each method to avoid misinterpretation and unnecessary waste of resources.

**Table 1:** Comparison of different acute ischemia modeling methods.

Modeling method	Induction approach	Strain	Age (weeks)	Sex	Ischemia recovery time	Complexity	Technical demand	Reference
Ligation/Electrocoagulation	Single femoral artery electrocoagulation/ligation	C57BL/6 mice	10–12	Male	7 days	Simple	Moderate	(7, 8)
	Single common iliac artery electrocoagulation/ligation	C57BL/6 mice	10–12	Male	7 days	Simple	Moderate	(7)
	Double electrocoagulation of femoral and common iliac arteries	C57BL/6 mice	10–12	Male	28 days, recovery to 54% of baseline	Simple	Moderate	(7, 9)
	Femoral artery double electrocoagulation/ligation with transection	C57BL/6 mice	10–12	Male	21–28 days	Simple	Moderate	(7, 10)
	Femoral artery and vein double electrocoagulation/ligation with transection	C57BL/6 mice	12	Male	14 days, recovery to 48% of baseline	Simple	Moderate	(11, 12)
	Common iliac artery and vein ligation with transection	C57BL/6 mice	Young (12 weeks) vs. Aged (72 weeks)	Male	Young: 14 days, recovery to 60% of baseline; Aged: 14 days, recovery to 25% of baseline	Simple	Moderate	(13)
Interventional embolization	Embolic agent (hydrogel filament) at the aorto-popliteal/saphenous bifurcation	Lewis rats	10–12	Male	14 days, recovery to 88.6% of baseline	Complex	High	(4)
	Embolic agent (polyvinyl alcohol particles) in external iliac and distal arteries	Sprague–Dawley rats	10–12	Male	7 days: infarction rate of thigh muscle: 17.3%; calf muscle: 33.7%	Complex	High	(14)
	Embolic agent (N-butyl cyanoacrylate) in external iliac and distal arteries	Sprague–Dawley rats	10–12	Male	7 days: infarction rate of thigh muscle: 43.6%; calf muscle: 50.3%	Complex	High	(14)
Photochemical embolization	Photosensitizers (Erythrosin B, Rose Bengal)	ICR mice, Wistar rats, Sprague–Dawley rats	8–12	Male	Not reported	Complex	High	(16–18)
Chemical injury	Ferric chloride	C57BL/6 mice, P2Y12-deficient mice	Not reported	Not reported	21 days	Complex	High	(20, 21)
Physical injury	Guidewire-induced endothelial denudation	C57BL/6 mice	18–22	Male	Not reported	Complex	High	(22–24)
TCM microparticle embolization	*Bletilla striata* microparticles	Wistar rats	10–12	Both sexes	21 days	Simple	Moderate	(27–29)

### 3.1 Modeling methods

#### 3.1.1 Ligation/electrocoagulation/resection

Arterial ligation is currently the most widely used approach for PAD model establishment. Based on the ligation technique, it can be categorized into nylon suture ligation and electrocoagulation occlusion. According to the number of ligations, it can be further divided into single ligation and double ligation, namely simple femoral artery ligation, or combined femoral and common iliac artery ligation. After ligation, the procedure may or may not involve transection of the targeted vessel; transection is more suitable for evaluating angiogenesis in distal ischemic tissues.

**Figure 2:**
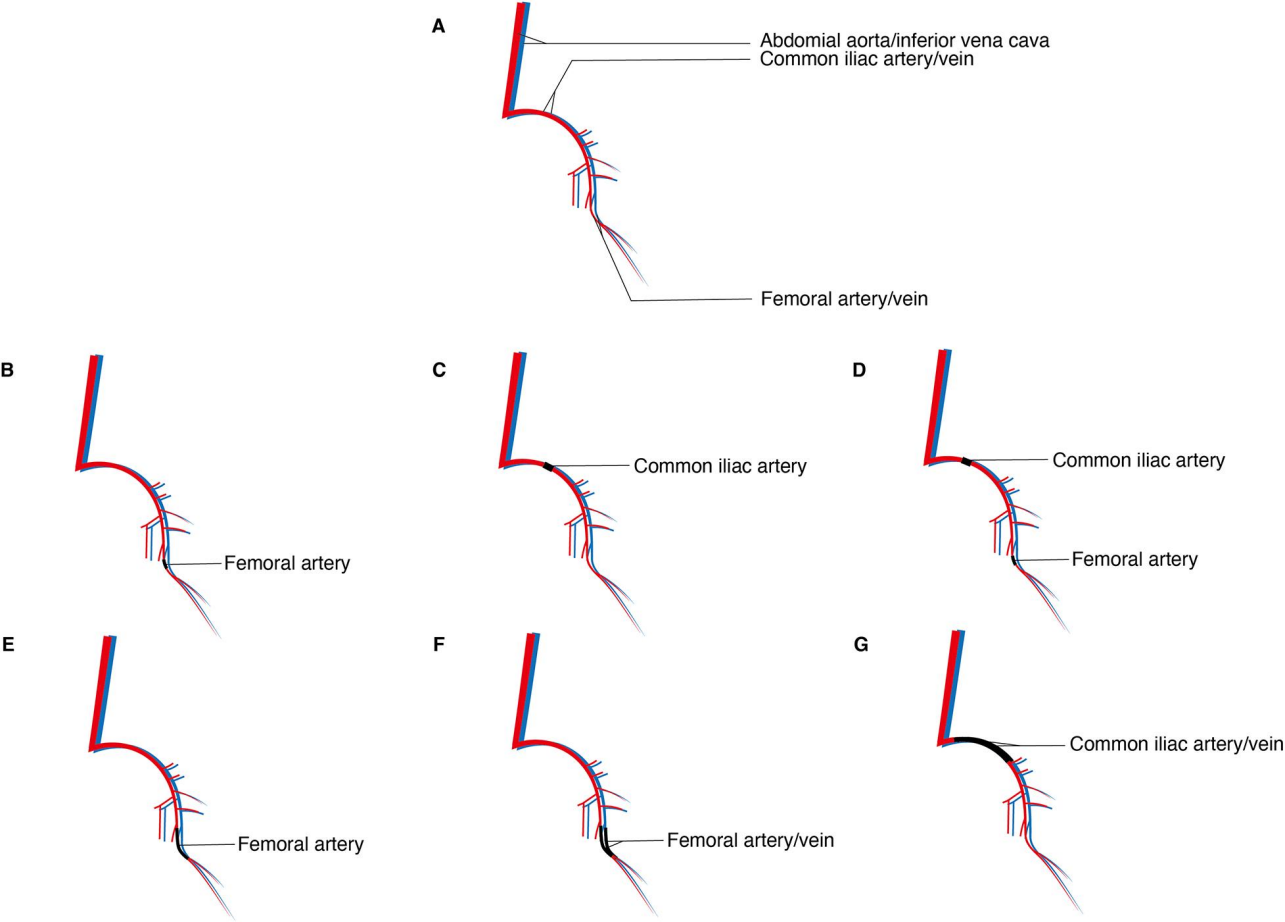
Different ligation/electrocoagulation methods; **(A)** sketch of the arteries and veins of the hindlimbs of the murine; **(B)** single femoral artery electrocoagulation/ligation; **(C)** single common iliac artery electrocoagulation/ligation; **(D)** combined femoral artery and common iliac artery double electrocoagulation; **(E)** femoral artery double electrocoagulation/ligation with transection; **(F)** femoral artery and vein double electrocoagulation/ligation with transection; **(G)** external iliac artery and vein double electrocoagulation/ligation with transection.

Considering these variations, murine hindlimb arterial ligation models can be classified into the following types: single femoral artery electrocoagulation/ligation (7, 8); single common iliac artery electrocoagulation/ligation (7); combined femoral artery and common iliac artery double electrocoagulation (7, 9); femoral artery double electrocoagulation/ligation with transection (7, 10); femoral artery and vein double electrocoagulation/ligation with transection (11, 12); and external iliac artery and vein double electrocoagulation/ligation with transection (13) (Figure 2).

The surgical procedures among these methods are generally similar. Following standard disinfection and anesthesia, the abdominal cavity or a unilateral inguinal region is incised to expose the target vessel for the designated procedure, while the contralateral side is used as a control. Observation periods, treatment durations, and reperfusion times differ across modeling techniques. Therefore, investigators should design their protocols according to specific experimental objectives, conduct pilot studies for validation, and thereby minimize unnecessary waste of time and experimental resources.

#### 3.1.2 Interventional embolization

During the ligation procedure, exposure, dissection, and ligation of the target artery often cause collateral damage to adjacent veins and neural networks, along with increased infiltration of inflammatory cells, which may compromise experimental outcomes. In contrast, interventional techniques exert relatively minor local effects and thereby expand the available approaches for establishing hindlimb ischemia models.

The interventional embolization method involves advancing a catheter through the common carotid artery of the rat/mice into the region between the iliac artery and the superficial caudal epigastric artery under angiographic guidance, followed by injection of embolic agents to occlude the common iliac artery, internal iliac artery, external iliac artery, femoral artery, popliteal artery, or saphenous artery, thereby inducing tissue ischemia (14). Currently used embolic agents include hydrogel filaments (4), polyvinyl alcohol (PVA) particles, and N-butyl cyanoacrylate (NBCA) (14), among which hydrogel filaments are the most widely applied.

#### 3.1.3 Photochemical embolization

Photochemical embolization is a method of inducing hindlimb ischemia through vascular endothelial dysfunction and localized thrombosis triggered by a photochemical reaction between a photosensitizer and focused illumination (15). Commonly used photosensitizers include Rose Bengal and Erythrosin B. After intravenous administration of the photosensitizer, the target artery is irradiated with filtered green light or cold white light for 30–40 min to induce thrombus formation (16–18). This technique represents a noninvasive approach to vascular occlusion, as it does not require surgical incision to generate intravascular thrombosis. Moreover, no significant bleeding, infarction, or widespread pro-apoptotic stimulation has been observed, indicating that this method allows for precise, region-specific occlusion without major side effects (18).

#### 3.1.4 Chemical injury

Kurz et al. (19) first established and validated a rat model of arterial injury and thrombosis induced by topical application of ferric chloride solution, and demonstrated that the resulting thrombus consisted of platelets and red blood cells interwoven within a fibrin network, resembling the characteristics of arterial thrombosis. Ohno et al. (20) subsequently applied the ferric chloride model to murine hindlimb arteries. Under a dissecting microscope, filter paper (approximately 1 × 2 mm) soaked in 10%–50% ferric chloride solution was placed directly on the target artery for 3–10 min, followed by removal, leading to gradual thrombus formation and eventual vascular occlusion. Previous studies have primarily employed P2Y12 knockout mice (20) and leukocyte-associated immunoglobulin-like receptor knockout mice (21) in this model.

#### 3.1.5 Physical injury

The physical injury method induces ischemia by mechanically damaging the arterial wall, thereby triggering platelet adhesion and mural thrombus formation. This is achieved by rotating a flexible guidewire within the vessel lumen to disrupt the endothelium, which subsequently promotes thrombus development (22, 23). Loss and necrosis of vascular smooth muscle cells in the arterial media occur as early as day 1 after injury. Over the following 14 days, thrombus formation and platelet deposition decline progressively, from nearly 100% on day 1 to approximately 25%. This model is commonly applied in transgenic mice deficient in protease-activated receptor-1, and is particularly useful for studying cell proliferation and medial thickening (24).

#### 3.1.6 Embolization with TCM microparticles

*Bletilla striata* embolic agents are prepared from the dried tubers of *Bletilla striata*. Their active component, mucilage, is a high-molecular-weight compound composed of four mannose molecules and one glucose molecule, and is known to exert anti-inflammatory, antitumor, and pro-coagulant effects (25, 26). In 1998, it was first developed as a vascular embolic agent for hepatic artery embolization studies (26); in 2005, Bletilla microparticle embolization was for the first time applied in establishing a rat hindlimb ischemia model (27). The procedure is performed under standard disinfection and adequate anesthesia. After isolating the femoral artery, the main trunk is clamped, and sterilized syringes are used to inject Bletilla microparticles into the artery. The clamp is then released, hemostasis is achieved by compression, and the skin is sutured (28, 29). Previous studies have demonstrated that Bletilla microparticle embolization achieves uniform and complete occlusion of hindlimb vessels in rats, effectively preventing the development of collateral circulation. In addition, it promotes erythrocyte aggregation, shortens coagulation time, and leads to secondary thrombus formation (27).

### 3.2 Comparison of modeling methods

The murine hindlimb arterial ligation model is the most widely used model of PAD and has numerous advantages. The procedure is simple, cost-effective, and technically well-established. Detailed experimental protocols can be found in previously published studies. Compared with coronary artery models, PAD surgery involves distal limb vessels, resulting in lower mortality and relatively safer operations for animals (5). However, as an invasive approach, local inflammatory responses are inevitable. Avoiding injury to surrounding nerves and veins requires considerable surgical skill. Surgical trauma induces the release of large amounts of growth factors and recruitment of monocytes, which further stimulate arteriogenesis via pro- and anti-inflammatory cytokines. This may mediate endothelial cell activation, migration, proliferation, and apoptosis, thereby influencing experimental accuracy (30). Nerves are a major source of vascular endothelial growth factors (VEGF), and their injury during surgery can impair angiogenesis. Indeed, experimental evidence suggests that nerves actively promote angiogenesis and arteriogenesis (31). Minor bleeding from small vessels during surgery can usually be controlled by compression, whereas injury to large vessels often necessitates proximal ligation, which alters circulation in the ischemic limb and compromises data accuracy. In some procedures, arteries and veins are ligated together, which not only prevents inadvertent venous injury but also enhances ischemic severity and reproducibility (8).

Interventional embolization and photochemical embolization avoid local inflammation and collateral tissue injury, thereby reducing the risk of infection. However, they require stricter laboratory conditions, greater technical expertise, and higher financial costs. Interventional embolization presents a steep technical barrier: researchers must be familiar with rat/mice vascular anatomy, the procedure is time-consuming, and radiation exposure poses additional risks. Photochemically induced thrombosis closely resembles lower limb arterial embolization. In ligation models, hindlimb circulation typically improves within 7 days (7); moreover, inflammation-driven arteriogenesis introduces confounding factors when studying angiogenesis and collateral circulation. In contrast, photochemical embolization produces more severe ischemia, leading to tissue discoloration, gangrene, and limb loss, closely mimicking the clinical disease course. This model allows fine-tuned control of variables—light intensity, exposure duration, irradiation site, and photosensitizer concentration—offering high flexibility. Furthermore, photosensitizers have no toxic effects on target organs, and animal survival rates are high (16). Nevertheless, the method can cause irreversible thrombotic occlusion and free radical generation, damaging vascular walls at the irradiation site. As no standardized parameters exist for light intensity or exposure time, researchers must determine optimal conditions empirically.

The chemical injury model induces arterial thrombosis via ferric chloride application, thereby avoiding vessel transection or ligation and the associated collateral tissue injury. This method does not require specialized equipment to induce thrombosis. The thrombi formed are platelet-rich and, under a dissecting microscope, closely resemble human arterial thrombi (32).

The physical injury method is currently more commonly used in carotid artery ischemia models (24). Its application to hindlimb ischemia requires further evaluation and the establishment of standardized protocols to improve reproducibility.

TCM microparticle embolization combines both physical and chemical approaches. *Bletilla striata* particles induce thrombosis pharmacologically while simultaneously blocking blood flow mechanically, thereby producing limb ischemia. The particles distribute evenly within vessels; in larger arteries, they may “migrate” under hemodynamic force into smaller branches, achieving uniform and complete embolization and effectively preventing collateral circulation. The particles slowly swell in blood, and their fibrous components resist absorption and degradation, exerting a sustained mechanical occlusive effect (27–29). Previous studies have shown that ischemia induced by Bletilla embolization can last 5 weeks to ∼2 months, offering long-lasting, stable, and reproducible outcomes (26, 27). However, the degree of embolization is difficult to quantify, and the method remains rarely applied. Moreover, the amount of granulation tissue available from this model is limited, which complicates studies on angiogenesis-targeted therapies (29).

## 4 Subacute–chronic ischemia models

PAD is a long-term chronic ischemic condition of the lower extremities, and acute ischemia models fail to fully recapitulate its pathological course. Subacute ischemia models prolong the duration of ischemia, thereby better simulating the chronic ischemic process in humans. Moreover, in subacute–chronic ischemia, the increase in shear stress occurs gradually rather than abruptly, providing more time for the adaptation and development of collateral arteries. Currently, modeling approaches include the Ameroid constrictor method, suture ligation, high-fat diet combined with suture ligation, anticoagulant silicone tube method, and the loop ligature technique (Table 2).

**Table 2:** Comparison of different subacute ischemia modeling methods.

Modeling method	Induction approach	Strain	Age (weeks)	Sex	Ischemia recovery time	Complexity	Technical demand	Reference
Ameroid constrictor	Ameroid constrictor	C57BL/6 mice, SD rats	10–12	Male	4–5 weeks	Relatively complex	High	(35–37)
Ligation method	Prolene suture	Lewis rats	4	Male	49 days to 70% of contralateral limb	Simple	Moderate	(38, 39)
High-fat Diet + ligation	High-fat diet + Prolene suture	SD rats	2	Male	42 days to 60% of contralateral limb	Simple	Moderate	(40–42)
Anticoagulant silicone tube	Anticoagulant silicone tube	SD rats	12	Male	28 days to 67% of baseline	Simple	Moderate	(43)
Ring method	Infusion tube ring	SD rats	Not specified	Both sexes	Not specified	Simple	Moderate	(44)

### 4.1 Modeling methods

#### 4.1.1 Ameroid constrictor method

**Figure 3:**
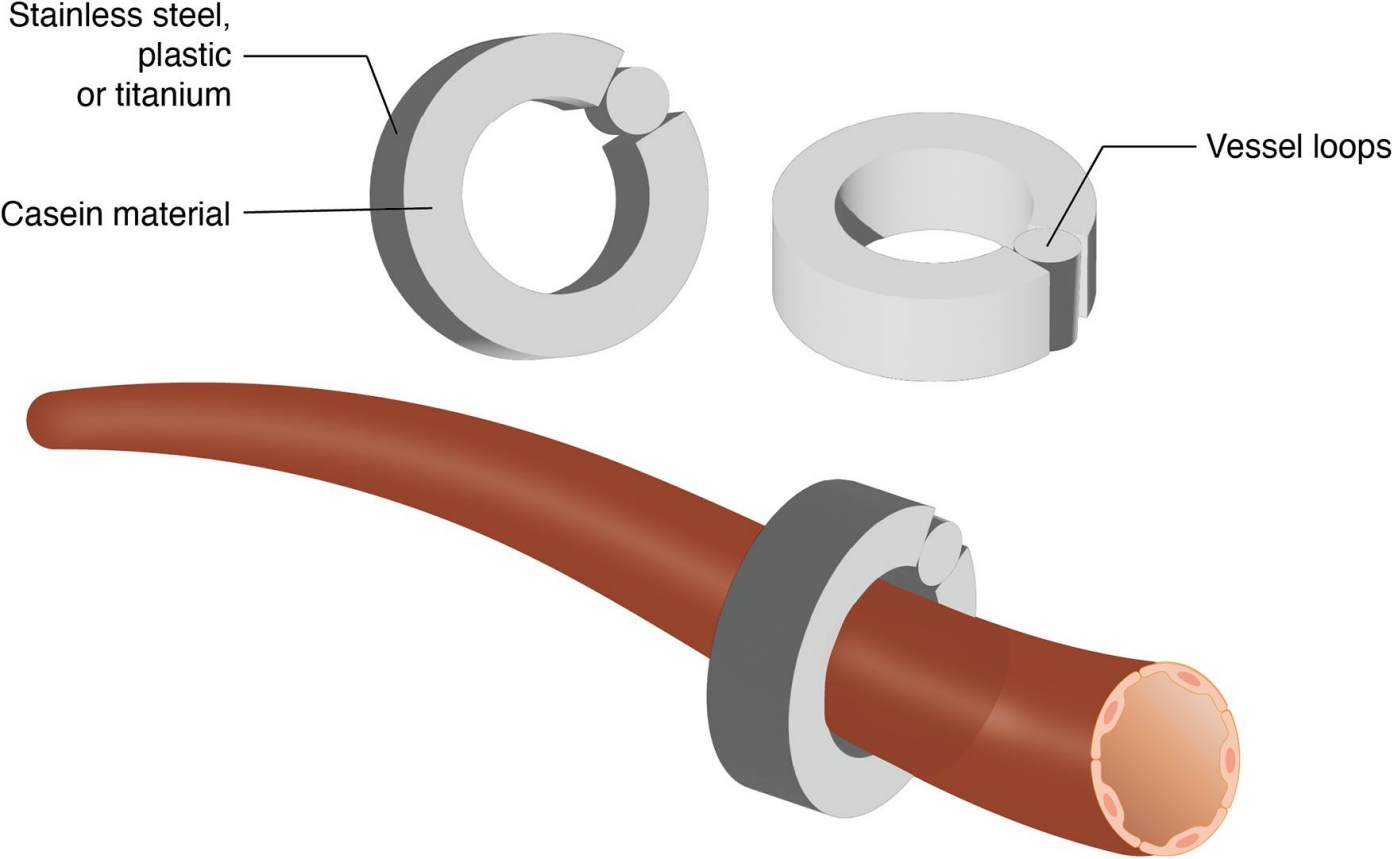
Schematic representation of ameroid constrictor placement on artery.

The Ameroid constrictor has been used in the coronary arteries of pigs and dogs to model atherosclerotic coronary artery disease (33, 34). Gale et al. (35) were the first to apply the Ameroid constrictor to a rat hindlimb ischemia model. The Ameroid constrictor consists of a stainless steel outer casing that encases an inner ring of casein, a hygroscopic material. Upon absorbing body fluids, the casein gradually swells, and the rigid stainless steel shell forces the casein to expand inward, ultimately occluding blood flow in the target artery (35, 36). The modeling procedure involves exposing and dissecting the target artery, selecting an appropriately sized Ameroid constrictor, and securing it around the vessel to complete the model (Figure 3) (37).

#### 4.1.2 Suture-Induced occlusion method

The suture-induced occlusion model, developed by the research team of Shengjia Yang (38, 39) is a chronic ischemia model that uses a foreign-body reaction caused by an intravascular suture to gradually induce vascular occlusion. The procedure is as follows: after adequate anesthesia, the femoral artery is isolated and incised. A 4-0 Prolene suture is introduced into the distal segment of the femoral artery up to the knee joint under a surgical fiberscope, after which the arterial incision is closed. The intraluminal suture causes mechanical narrowing of the artery without immediate interruption of blood flow. As a foreign-body reaction develops, intimal hyperplasia ensues, leading to gradual occlusion and sustained hindlimb ischemia. This method maintains ischemia in the rat hindlimb for more than 6–7 weeks, with relatively low incidence of acute muscle necrosis and fibrosis (38).

#### 4.1.3 High-fat diet combined with suture-induced occlusion

Chen Lei et al. (40) noted that although the suture-induced occlusion method provides a robust chronic ischemia model, surgical trauma and disease burden may shorten the survival of experimental animals, resulting in insufficient sample size. To develop a model of chronic limb ischemia with a shorter induction period, they modified Yang's method by feeding the animals a high-fat diet for two weeks before surgery. This dietary intervention significantly elevated serum lipid levels in the experimental group compared to controls, enhancing the foreign-body response to the intravascular suture, promoting intimal hyperplasia, and accelerating model establishment. Similarly, Emma Fletcher et al. (41, 42) successfully induced chronic hindlimb ischemia in C57BL/6J mice as a PAD model using a 16-week high-fat/high-sucrose diet combined with left femoral artery ligation.

#### 4.1.4 Anticoagulant silicone tube method

Yingbin Jia's research group (43) developed a chronic ischemia model using an anticoagulant silicone tube. The procedure is as follows: after adequate anesthesia, the iliacofemoral artery is exposed, and side branches including the superficial iliac circumflex, femoral profound, external pudendal, and descending genicular arteries are ligated and transected with 7-0 atraumatic sutures. After temporarily clamping the bifurcation of the external iliac, saphenous, and popliteal arteries, one-third of the anterior wall of the femoral artery is incised. The lumen is flushed with heparinized saline, and an 8 mm-long silicone tube (inner diameter 0.6 mm) is inserted via the femoral incision. The tube is positioned to cover the arterial opening and secured with 7-0 atraumatic sutures. This setup mimics the process of chronic thrombus formation within the tube, simulating clinical peripheral arterial occlusive disease.

#### 4.1.5 Perivascular cuff method

Yimin Fan's research group (44) established a chronic ischemia model using a perivascular cuff made from an intravenous infusion tube. The procedure is as follows: after adequate anesthesia, the skin is incised along the femoral sheath, and the femoral artery is isolated. A 5 mm-long plastic infusion tube (inner diameter 1 mm) is longitudinally cut to form a cuff, which is placed around the femoral artery. The cuff is secured in place with sutures, with tension adjusted according to vessel diameter, thereby creating a chronic hindlimb ischemia model.

### 4.2 Comparison of modeling methods

The Ameroid constrictor method induces chronic hindlimb ischemia through progressive compression of the target vessel, as the casein layer within the constrictor absorbs interstitial fluid and gradually expands. Ameroid constrictor-induced occlusion in mice results in relatively low shear stress–dependent and inflammatory gene expression, with limited blood flow recovery at 4–5 weeks postoperatively and minimal muscle necrosis (45). This method requires only surgical exposure and isolation of the target artery before placement of the constrictor, thereby minimizing local injury and inflammatory interference with angiogenesis, and is considered a reliable approach for establishing subacute ischemia models. However, several limitations exist. First, Ameroid constrictors are more costly compared to conventional arterial ligation. Second, if the groove of the constrictor is too shallow, the artery may slip out during constriction, leading to modeling failure. Third, uneven casein distribution within the constrictor may accelerate occlusion, negating the intended chronic process (37). Finally, the gradual occlusion reduces shear stress and prolongs collateral and distal angiogenesis, posing significant challenges to animal survival. Therefore, further refinement and optimization of this model are still required.

The suture-embolization method can maintain hindlimb ischemia for an extended period, while the pathological changes and dynamic recovery of blood flow better resemble the clinical course of chronic ischemia, making it a solid platform for evaluating therapeutic interventions. Neointimal hyperplasia appears as early as postoperative day 14, and by day 42, the artery is nearly occluded due to extensive neointimal proliferation. Interestingly, acute ischemic manifestations such as pallor, pulselessness, paresthesia, paralysis, and pain (“5 Ps”) are rarely observed; instead, muscle atrophy is evident during the late phase (42–49 days), consistent with clinical findings (38). However, since most PAD patients present with hyperlipidemia, standard rats may not fully replicate the chronic ischemia seen in humans with elevated lipid levels. Furthermore, as the method requires femoral arteriotomy, how to minimize local inflammatory infiltration and shear stress remains an issue for further investigation.

The high-fat diet combined ligation method allows for more rapid establishment of an ischemic model. Decreases in muscle strength occur earlier compared to controls, and muscle function, Doppler, and histological findings confirm more severe and sustained ischemia in the experimental group (40). Nonetheless, as chronic ischemia is inherently a long-term process, the accelerated onset of ischemia raises concerns regarding whether it induces artificially elevated shear stress. Moreover, earlier inflammatory cell infiltration observed in the experimental group may be detrimental to the fidelity of chronic ischemia modeling.

In the anticoagulant silicone tube model, branches of the external iliac artery—including the superficial circumflex iliac artery, deep femoral artery, and other collaterals—are ligated or divided, along with all branches of the femoral artery segment. This effectively blocks collateral flow and sustains hindlimb ischemia. Results show that blood flow in the ischemic limb reaches its lowest point at 7 days postoperatively. Although this does not replicate the years-long course of severe chronic ischemia seen clinically, it provides a significantly improved simulation compared to acute ischemia models (43). This method is technically simple, reproducible, associated with low mortality, and yields relatively stable ischemic severity. However, excessive interruption of collaterals, combined with femoral artery ischemia, may deviate from clinical reality.

The ring method is straightforward, low-cost, and technically feasible. However, displacement of the infusion tube ring may occur due to insufficient suture fixation, and the plastic material itself may cause local tissue injury, potentially confounding experimental outcomes. Therefore, the practical applicability of this method requires further validation.

## 5 I/R model

Acute lower limb ischemia is a common vascular emergency in clinical practice, characterized by a sudden reduction in blood supply to the limb, leading to intermittent claudication, tissue necrosis, and ulceration, which severely impair patients' quality of life (46, 47). Timely reperfusion is the cornerstone of treatment; however, it is often complicated by I/R injury, which causes vascular and muscular damage in the limb as well as multiple organ dysfunction syndromes involving the heart, lungs, liver, kidneys, and brain (48, 49). This complication is a major contributor to the high mortality associated with acute lower limb ischemia (47). To mitigate I/R injury, it is essential to establish and investigate ischemia/reperfusion and preconditioning models.

### 5.1 Modeling methods

The limb I/R model is typically established by first inducing acute ischemia by a tourniquet (50–52), pneumatic tourniquet (53), rubber band (54), orthodontic elastic band (55), aneurysm clip (56), non-traumatic microvascular clamp (51, 54, 57, 58), combined collateral cauterization and transection (58), or a tension cable system (59). These interventions are usually applied to the femoral or abdominal aorta and femoral artery ligation, thereby occluding blood flow to the lower limb for approximately 2–6 h (most commonly 3 h). Reperfusion is then achieved by removing the tourniquet or clamp, generally lasting 2–6 h.

Since I/R can cause severe injury to both the limb and internal organs, preconditioning strategies are often employed to mitigate reperfusion injury. For example, dexmedetomidine preconditioning has been shown to protect the liver and lungs against lower limb I/R injury (54, 60); local administration of DNase I and intravenous administration of a moderate dose of seviprotim (15 mg/kg bw/day) effectively eliminate neutrophil extracellular traps generated during reperfusion injury, thereby improving perfusion, promoting angiogenesis, preserving hindlimb function, and preventing muscle fibrosis (53); meclofenamate significantly alleviates skeletal muscle injury caused by lower limb I/R (51).

### 5.2 Evaluation

The modeling methods for I/R are relatively standardized, with the main point of debate concerning the duration of “ischemia” and “reperfusion” during the process. Previous studies have reported ischemia and reperfusion times ranging mostly from 2 to 6 h (54, 57, 61). Evidence indicates that ischemia lasting 3 h causes significant, yet localized and reversible injury, suggesting that “3 h of ischemia followed by 3 h of reperfusion” more closely mimics clinical scenarios and can therefore serve as a representative I/R model (50, 59). Although the I/R model is technically straightforward, strategies to mitigate reperfusion injury warrant further investigation. While numerous studies have demonstrated the protective role of preconditioning against I/R-induced injury in target organs, clinical translation remains limited, highlighting a critical direction for future research. Additionally, studies have shown that the severity of reperfusion injury does not differ among tissues examined after lower-limb I/R (62); thus, changes in each vital organ should be carefully monitored.

Although I/R causes tissue damage, appropriately applied remote ischemic preconditioning may have beneficial effects. Remote ischemic preconditioning refers to transient, non-lethal cycles of ischemia and reperfusion in the arm or leg that can protect against endothelial dysfunction induced by sustained ischemia and reperfusion (63, 64). For example, reversible and short-term lower-limb I/R (four cycles of 5 min of ischemia followed by 5 min of reperfusion) can provide substantial cardioprotection, significantly reducing myocardial IR injury (65, 66). Therefore, the dual-edged role of I/R warrants in-depth investigation.

## 6 Influencing factors

Different modeling methods may affect experimental outcomes; however, factors such as animal strain, genetics, sex, age, and anesthetic approach should also be carefully considered.

### 6.1 Strain

Helisch et al. (67) investigated C57BL/6, BALB/c, and 129S2/Sv mice following femoral artery ligation, assessing blood perfusion, collateral formation, and exercise tolerance on days 0, 7, 14, 21, and 28. Their results demonstrated that genetic differences in pre-existing collateral vascular systems influence collateral artery growth and the tissue environment after femoral artery occlusion. Other studies similarly found that, compared with BALB/c mice, C57BL/6 mice possess a more developed pre-existing collateral network and exhibit greater arteriogenesis in hindlimb ischemia models (68, 69). These findings suggest that different strains exhibit distinct blood perfusion recovery patterns, warranting stratified comparisons.

### 6.2 Genetics

Eugenio et al. (70) compared C57BL/6 mice with CD4-deficient mice after left femoral artery ligation. C57BL/6 mice exhibited superior hindlimb perfusion and collateral circulation compared with CD4-deficient mice. When CD4T cells from the spleen were transfused into CD4-deficient mice, the exogenous CD4 cells selectively homed to ischemic infiltrated regions within 24 h, accompanied by enhanced macrophage infiltration and VEGF expression. Consequently, blood flow recovery in the CD4 group resembled that of C57BL/6 mice. The same group further demonstrated that, after femoral artery ligation, CD8+ T cells infiltrated collateral growth sites and recruited CD4+ monocytes via IL-16 expression, underscoring the crucial role of the immune system in modulating collateral development in response to peripheral ischemia (71). Therefore, immune deficiencies in mice may confound experimental results.

### 6.3 Sex

Peng et al. (72) reported that female C57BL/6J mice exhibited slower perfusion recovery than males 7 days after femoral artery ligation, likely due to reduced collateral remodeling, impaired angiogenesis, and endothelial dysfunction. Sieveking et al. (73) further showed that androgens exert sex-specific effects on angiogenesis, with endogenous androgens promoting neovascularization in ischemic injury. Thus, sex differences should be considered as an influencing factor in model outcomes.

### 6.4 Age

Zhuo et al. (74) found that juvenile (6–8 weeks) mice demonstrated faster and more complete perfusion recovery than aged (60–64 weeks) mice. Accordingly, aged mice may be preferable for studies on pro-angiogenic interventions, as they allow clearer observation of treatment effects, whereas juvenile mice may be better suited for studies targeting anti-angiogenic processes. The inclusion of animals of different ages adds diversity and depth to experimental design.

### 6.5 Anesthetic

The choice of anesthesia can also influence modeling. Studies have shown that α-adrenergic agonists may induce early peripheral vasoconstriction, thereby interfering with experimental outcomes (75). Furthermore, the combined use of ketamine and α-agonists is not suitable for studies involving vascular systems rich in smooth muscle cells (76).

## 7 Murine hindlimb ischemia models in the context of atherosclerosis

The standard hindlimb ischemia models are typically established in healthy, young mice. However, clinical PAD predominantly occurs in aged individuals with underlying comorbidities, most notably atherosclerosis. To better simulate this clinical context, researchers have increasingly employed genetically modified mice, such as the apolipoprotein E knockout (ApoE^−^/^−^) and the low-density lipoprotein receptor knockout (LDLR^−^/^−^) mice.

When fed a high-fat diet, these mice develop systemic hypercholesterolemia and atherosclerosis. The use of such models in hindlimb ischemia studies allows for the investigation of PAD in a metabolically and vasculopathically compromised environment (77). Evidence suggests that the presence of atherosclerosis can significantly impair post-ischemic angiogenesis and arteriogenesis (78), leading to delayed perfusion recovery and more severe functional impairment compared to wild-type controls (79). Plasma hypercholesterolemia is antiangiogenic due to elevated levels of low-density lipoprotein (80).

While these models do not directly simulate “cardiac involvement” in the sense of primary myocardial pathology, the systemic atherosclerotic burden they develop does affect the coronary arteries, making them excellent models for studying the common co-morbidity of PAD and coronary artery disease. Therefore, for studies aiming to test therapeutic interventions for PAD in a clinically relevant setting, the ApoE^−^/^−^ and LDLR^−^/^−^ models, particularly when combined with a high-fat diet, offer a superior platform that recapitulates the complex pathophysiology of the human disease.

## 8 Conclusion and perspectives

Overall, murine hindlimb ischemia models exhibit considerable diversity, allowing researchers to tailor their choice of model according to specific experimental goals and conditions. Acute ischemia models are the most widely studied, with relatively mature protocols that meet most current research needs. By contrast, subacute and chronic ischemia models remain less developed, requiring further refinement and innovation. While I/R models are relatively easy to establish, strategies to minimize reperfusion injury continue to represent a research hotspot. Importantly, leveraging the beneficial aspects of I/R in preventing or treating other diseases holds significant potential for future clinical applications (Table 3).

**Table 3:** Summary of advantages and limitations of major murine hindlimb ischemia models.

Modeling type	Advantages	Limitations	Sample size	Mortality/Key safety notes	Reference
Single femoral artery electrocoagulation/ligation	(1) The surgical procedure is simple and rapid; (2) The model exhibits high standardization and excellent reproducibility; (3) The inflammatory response following ischemia is relatively mild; (4) Blood flow recovers quickly (within 7–14 days) in strains such as C57BL/6, making it suitable for studying spontaneous recovery mechanisms.	(1) The treatment window is short, making it difficult to observe the therapeutic effects of interventions in strains with rapid recovery; (2) The degree of ischemia is strain-dependent and may be insufficient to induce a robust angiogenic response in certain strains.	10–15 per group	Not reported	(7, 8)
Single common iliac artery electrocoagulation/ligation	(1) Relatively simple procedure, performed via retroperitoneal approach; (2) Controllable ischemia level, effectively reducing distal blood flow.	(1) Compared to single femoral artery electrocoagulation, no significant advantage was demonstrated in blood flow recovery speed or pattern; (2) The therapeutic window remains similarly brief (rapid recovery within 7 days).	9 per group (in comparative method study)	Not reported	(7)
Double electrocoagulation of femoral and common iliac arteries	(1) Simulates clinically relevant multi-segmental lesions with more severe and prolonged ischemia; (2) Creates a significantly extended therapeutic window (e.g., only 54% recovery after 28 days), making it ideal for evaluating the efficacy of pro-angiogenic therapies or cell therapies; (3) Equally effective in immunodeficient mice, suitable for studies using human cells.	(1) More severe ischemia increases the risk of tissue necrosis (e.g., toe necrosis); (2) Surgery involves two sites and is slightly longer; (3) Not suitable for studying arterialization of specific pre-existing collaterals originating from the deep femoral branch, as proximal iliac artery flow is also blocked.	9–22 per group	Approximately 30% (3/10) of C57BL/6 mice developed one or more cases of nail necrosis; in immunodeficient mice, the incidence of nail necrosis reached up to 90% (9/10). No deaths were reported.	(7, 9)
Femoral artery double electrocoagulation/ligation with transection	(1) Induces severe and stable ischemia, eliminating the possibility of vascular recanalization; (2) Effectively stimulates a robust angiogenesis response, serving as a classic model for studying this process.	(1) It involves significant surgical trauma and is more complex and time-consuming to perform; (2) Blood flow restoration relies on extensive connections within the newly formed capillary network rather than typical pre-existing collateral enlargement (arteriogenesis). Angiographic patterns appear disorganized, lacking the characteristic “spiral” collaterals; (3) Not suitable for studies specifically investigating arteriogenesis.	Each group of animals used for analysis at different time points must include ≥3 animals.	Approximately 10% of mice exhibited toe necrosis during the first postoperative week, which subsequently healed. No mortality was reported.	(10)
Femoral artery and vein double electrocoagulation/ligation with transection	Recommended Models: A-strip group (arterial stripping): Produces relatively severe and stable ischemia (9/10 cases with toe necrosis), suitable as a stable severe ischemia model. Group A (Arterial Transection): Simple procedure, mild and chronic ischemia (7/10 without necrosis, 3/10 with toe necrosis). Suitable as a chronic mild ischemia model, more closely resembling the clinical presentation in most patients.	(1) Uneven Ischemia Severity: Even within the same method group, ischemic injury severity (ranging from no necrosis to knee necrosis) exhibits individual variability and instability, particularly pronounced in the Prox-A and AV-strip groups; (2) A-strip surgery is more complex: Requires dissection of an arterial segment, making the procedure more time-consuming than simple ligation or transection; (3) Interference from venous ligation: The AV group introduces venous reflux obstruction, whose pathological changes do not correspond to pure arterial disease.	6–10 per group	Not reported	(11, 12)
Common iliac artery and vein ligation with transection	(1) Non-ischemic limb incision: The surgical incision is made in the abdomen, avoiding the creation of a surgical wound on the ischemic limb. This eliminates interference from wound healing on local inflammation and stem cell homing, allowing for a purer study of the effects of ischemia itself; (2) Simulates severe ischemia: The ligation site is positioned closer to the cardiac end (common iliac artery), blocking more collateral sources and potentially inducing more severe ischemia than simple femoral artery ligation alone; (3) Reveals age differences: Successfully used to compare differences in blood flow recovery, function, and angiogenesis/arteriogenesis responses between young and aged mice following severe ischemia.	(1) Simultaneous venous ligation: Ligation of the iliac vein to enhance technical success rates and ischemia severity distinguishes this model from clinically common arterial occlusive diseases; (2) Distinct blood flow recovery pattern: Young mice exhibit 60% baseline recovery by day 14, with recovery kinetics potentially differing from other models; (3) Survival limitations in aged mice: Poor postoperative survival in aged mice restricts long-term (>2 weeks) observation.	15(Young)/20(Aged) per group	Not reported (poor postoperative survival in aged mice was mentioned)	(13)
Embolic agent (hydrogel filament) at the aortopopliteal/saphenous bifurcation	(1) Minimally invasive: Performed via catheterization, eliminating the need for incisions on the ischemic limb and maximally preserving tissue integrity (nerves, vessels, muscle bundles) in the surgical area; (2) Mild inflammatory response: Compared to open surgery, significantly reduced early inflammatory cell infiltration and release of inflammatory factors (e.g., IL-1α, IL-18), minimizing surgical trauma interference with angiogenesis studies; (3) Clinical simulation: More closely mimics the pathophysiological processes of human atherosclerotic occlusion or embolism; (4) High reproducibility: Enables controlled, uniform, and repeatable vascular occlusion.	(1) Technically complex and demanding: Requires specialized interventional radiology skills, microsurgical equipment (binocular loupes), and real-time imaging guidance (C-arm); (2) Expensive equipment and materials: Requires specific microcatheters, guidewires, and embolization materials (e.g., hydrogel coils); (3) Risk of procedural failure: Risks include misembolization and vasospasm, leading to animal exclusion; (4) Slow early blood flow recovery: At 3 days post-procedure, blood flow recovery may be slower than in surgical models, potentially due to reduced inflammatory stimulation.	24 per group	Not reported	(4)
Embolic agent (polyvinyl alcohol particles/N-butyl cyanoacrylates) in external iliac and distal arteries	(1) Avoid surgical trauma: Unlike surgical resection, it does not create incision wounds, eliminating interference from the wound healing process; (2) Induce muscle infarction: Embolization with NBCA or PVA can cause distinct acute muscle infarction, suitable for studying infarction and subsequent changes; (3) No magnetic susceptibility artifacts: Unlike platinum coils, NBCA and PVA materials produce no magnetic susceptibility artifacts during MRI examinations, facilitating imaging assessment; (4) Simulate diverse clinical scenarios: Different embolization materials (dense NBCA distal embolization vs. PVA proximal embolization) can mimic distinct clinical ischemia patterns.	(1) High technical difficulty: Requires interventional expertise and dedicated imaging equipment; (2) Material properties influence outcomes: Different embolization materials (NBCA, PVA) produce varying degrees of ischemia and infarct size, necessitating careful selection based on study objectives; (3) Inflammatory factors not evaluated: The chemical and mechanical properties of different materials may trigger distinct cytokine releases, which were not assessed in this study.	4 per group	Not reported	(14)
Photosensitizers	(1) Non-invasive, non-mechanical: Induces thrombosis within blood vessels without surgical incisions, minimizing surgical trauma and inflammatory interference; (2) Highly reproducible and controllable: Precisely regulates ischemia location and severity by adjusting light intensity, exposure duration, spot position, and photosensitizer concentration; (3) Closer to human pathology: Simulates ischemia caused by thrombotic vascular occlusion, more closely resembling the mechanism of clinical peripheral arterial disease; (4) Suitable for molecular imaging: The absence of surgical incisions avoids background signal interference from incision inflammation, facilitating continuous and repeated molecular imaging monitoring.	(1) Requires specialized equipment: Depends on specific wavelength light sources (e.g., cool white light, green laser) and photosensitizers, presenting higher technical barriers and costs; (2) Relatively complex operation: Involves intravenous injection of photosensitizers, precise light positioning, and temporal control; (3) Potential phototoxicity: May cause free radical damage to vascular walls in irradiated areas; (4) Limited research history: Less extensively applied in limb ischemia models compared to traditional ligation methods; its long-term pathological progression and drug responses require further validation for human relevance.	8–15 per group	All survived until the end of the experiment. No tissue damage related to the photosensitizer or light exposure was observed.	(16–18)
Ferric chloride	(1) Effectively induces acute thrombosis: Rapidly and reliably forms occlusive thrombi in local arteries; (2) Simulates mild limb ischemia: Induces moderate ischemia typically without severe limb necrosis, closely mimicking the intermittent claudication experienced by most PAD patients; (3) Suitable for functional assessment: Post-ischemic gait abnormalities can be quantified using high-sensitivity systems (e.g., CatWalk).	(1) Invasive procedure: Requires surgical exposure of the vessel and placement of FeCl₃-impregnated filter paper; (2) Nonspecific chemical injury: FeCl₃ may cause direct chemical damage to perivascular tissues (including nerves), potentially confounding functional outcomes.	10 per group	Not reported	(20)
Guidewire induced endothelial denudation	(1) Closest to clinical balloon angioplasty, simulating the entire process including endothelial stripping, vascular wall stretching, platelet aggregation, and inflammatory cell infiltration; (2) Induces stable and reproducible neointimal hyperplasia, suitable for studying restenosis mechanisms; (3) With standardized techniques, skilled operators can complete the procedure within 20 min.	(1) Extremely high technical difficulty: Requires inserting a 0.38 mm diameter wire into a femoral artery branch approximately 0.1 mm in diameter, demanding meticulous manipulation; (2) Prone to intraoperative complications such as arterial perforation and major hemorrhage, which may result in animal mortality.	Not reported	The risk of intraoperative mortality is high, primarily due to massive hemorrhage caused by arterial perforation. While specific percentages are not reported in the literature, it is emphasized that “forceful insertion could result in loss of the mouse”.	(22–24)
Bletilla striata microparticles	(1) Effectively establishes a chronic persistent ischemia model with significant and sustained ischemic effects (lasting up to 2 months or longer); (2) Simulates the chronic arterial occlusion process, more closely approximating the pathological state of clinical ischemic diseases (compared to acute ligation); (3) The small particle size (∼47 μm) enables embolization of peripheral small arteries and arterioles, effectively blocking the establishment of collateral circulation.	(1) Complex preparation process for the embolization agent: Requires ultrafine grinding technology to produce Bletilla striata particles of a specific diameter (e.g., 47 μm); (2) Uneven degree of ischemia: Due to variations in the ability to establish collateral circulation among individual animals, tissue necrosis may occur to differing extents, affecting the standardization of tissue sampling; (3) Primarily validated in rat models; fewer reports on its application and optimization in mice.	5–8 per group	No animal deaths occurred during or after the procedure.	(27–29)
Ameroid constrictor	(1) Simulates chronic ischemia processes closer to clinical conditions; (2) Allows controlled occlusion rates to accommodate diverse research requirements; (3) Reduces acute inflammation and necrosis.	(1) High equipment costs and complex operational techniques; (2) Occlusion rates are influenced by device quality and environmental humidity; (3) Significant variability in responses across different mouse strains.	8–13 per group	No significant surgical mortality.	(35–37)
Ligation method	(1) Simple operation and low cost; (2) Can simulate chronic ischemia with prolonged ischemic duration.	(1) Mild ischemia with rapid recovery; (2) Limited collateral vessel opening and weak arterialization; (3) Primarily applicable to rats, with limited use in mice.	4–10 per group	No significant surgical mortality.	(38, 39)
High-fat diet + Ligation	(1) Simulates common risk factors for human PAD (high-fat diet, alcohol); (2) Ischemic manifestations appear early and recover slowly, making it suitable for chronic ischemia studies.	(1) Long experimental cycle (requires several weeks of feeding); (2) Dietary factors may introduce metabolic confounding variables; (3) Ischemia severity may vary due to individual differences.	12–20 per group	No significant surgical mortality.	(40–42)
Anticoagulant silicone tube	(1) Simulates the progressive thrombosis process, closely mimicking clinical chronic occlusion; (2) Allows controlled ischemia severity with gradual blood flow restoration; (3) Relatively simple to perform with high reproducibility.	(1) Requires prevention of silicone tube displacement or premature thrombus detachment; (2) Suitable for rats; challenging to perform in mice.	20 per group	Not reported	(43)
Ring method	(1) Simple to operate, capable of simulating chronic ischemia and applicable for pain research; (2) Suitable for drug intervention studies (e.g., nerve block).	(1) Degree of ischemia depends on ligature tightness, making standardization challenging; (2) May induce perivascular fibrosis rather than true occlusion; (3) Suitable for rats; difficult to perform on mice due to their smaller vessels.	13 per group	Not reported	(44)

Based on the reviewed literature, the acute ischemia model induced by simple femoral artery ligation appears to be the safest and most reproducible technique, with minimal reported mortality and low operational risk, making it suitable for most angiogenesis studies. In contrast, models that induce severe ischemia, such as double ligation with transection or photochemical embolism, are associated with higher rates of limb necrosis and auto-amputation, and should be employed with caution. The subacute-to-chronic models, while better mimicking human PAD, often involve more complex surgeries and longer ischemic durations, which can pose significant challenges to animal survival and welfare, necessitating careful perioperative management. The frequent underreporting of mortality data in the literature highlights an area for future improvement, and we strongly advocate for the standardized reporting of survival rates and perioperative complications in all future studies to facilitate better model selection.
